# Immune Dysregulation in Pediatric Common Variable Immunodeficiency: Implications for the Diagnostic Approach

**DOI:** 10.3389/fped.2022.855200

**Published:** 2022-03-23

**Authors:** Aleksandra Szczawińska-Popłonyk, Katarzyna Ta̧polska-Jóźwiak, Eyal Schwartzmann, Natalia Popłonyk

**Affiliations:** ^1^Department of Pediatric Pneumonology, Allergy and Clinical Immunology, Institute of Pediatrics, Poznań University of Medical Sciences, Poznań, Poland; ^2^Poznan University of Medical Sciences, Poznań, Poland

**Keywords:** common variable immunodeficiency, immune dysregulation, autoimmunity, lymphoproliferation, malignancy, children

## Abstract

Infections and infectious complications are hallmarks of common variable immunodeficiency (CVID) and the leading cause of morbidity and mortality in affected patients at any age. However, the pediatric CVID is no longer perceived as a primary immunodeficiency associated solely with infectious manifestations; autoimmune, allergic, lymphoproliferative, and malignant disorders and organ-specific immunopathology also characterize the spectrum of non-infectious complications. In this study, we sought to determine the role of immune dysregulation and frequency of non-infectious sequelae in children affected with CVID. We also aimed at providing an insight into the pathogenesis of non-infectious complications and at delineating the diagnostic approach to pediatric CVID with immune dysregulation. An in-depth retrospective analysis of clinical manifestations and their correlations with selected immune parameters was performed in a group of 39 CVID children, followed by our pediatric immunology department. Whereas recurrent sinopulmonary infections were present in all (100%) of the children studied, an unexpectedly high rate of non-infectious disorders and immune dysregulation phenotypes were observed in as many as 32 (82.05%) patients, compared with infection-only phenotypes limited to 7 (17.95%) male patients. The most common inflammatory comorbidity was asthma, diagnosed in 21 (53.85%) patients. The second most frequent immune dysregulation group was autoimmune disorders, present in 18 (46.15%) of the children studied with a high rate of autoimmune thyroiditis in as many as 10 (25.64%) of the CVID-affected children. Lymphoproliferation was seen in 14 children (35.90%), and, among them, lymphadenopathy occurred in nine (23.08%) cases and granulomatous lymphocytic interstitial lung disease in seven (17.95%) cases. Finally, malignancies occurred in two female patients (5.13%), papillary thyroid cancer in the first one and T-cell lymphoblastic leukemia in the other one. The most prominent abnormalities in the B- and T-cell compartment contributing to complex immune deficiency and immune dysregulation phenotypes were seen in the autoimmunity group, showing significant reductions in the switched memory B cell, naive T helper cell, and regulatory T-cell subsets. Herein, we document the previously unreported high rate of immune dysregulation in pediatric CVID as a clinical and diagnostic challenge with the variability of defects in the humoral and cellular immune responses.

## Introduction

Common variable immunodeficiency disorders (CVIDs) are a heterogeneous group of inborn errors of immunity (IEIs) characterized primarily by deficiency of antibody production as their common denominator. Low serum immunoglobulin levels, dysfunctional specific antibody response along with lymphocyte developmental and functional abnormalities, and innate immune defects make affected children susceptible to infections. Recurrent respiratory tract infections, such as otitis media, sinusitis, bronchitis, and pneumonia, and gastrointestinal infections are the most frequent clinical presentation of pediatric CVID (1, 2). Whereas, infections and infectious complications are hallmarks of CVID and the leading cause of morbidity and mortality in affected patients at any age (3), autoimmune, allergic, lymphoproliferative, and malignant disorders and organ-specific immunopathology characterize the spectrum of non-infectious complications related to pediatric CVID (1). Hence, CVID is no longer perceived as a primary immunodeficiency associated solely with concomitant infectious manifestations (4). The role of non-infectious disorders defining CVID has also been highlighted in the 2016 International Consensus Document (ICON) (5, 6) and 2019 European Society for Immunodeficiencies (ESID) Registry working definitions for the clinical diagnosis of IEIs (7). The wide range of CVID-related disorders encountered in affected children includes polyclonal lymphoproliferative disease in the form of granulomatous lymphocytic interstitial lung disease (GLILD) (8, 9) and intracranial granulomas (10) along with multiorgan involvement of the lymph nodes, spleen, liver, eye, parotid gland, and skin. The increasingly recognized features of immune dysregulation also comprise autoimmune disorders, which may be the first and the sole clinical presentation of CVID. The broad constellation of phenotypic features includes autoimmune cytopenias, such as autoimmune hemolytic anemia (AIHA) and autoimmune thrombocytopenic purpura (ITP), arthritis, and gastrointestinal disorders, such as colitis, gastritis, celiac disease, and endocrinopathies (11). The diagnosis of asthma is frequently established in CVID children; its frequency has been estimated in pediatric case series to range from 66% (1) to 83% (12). However, the diagnosis of asthma in a large proportion of reported cases precedes the diagnosis of CVID, and the infection-induced obstructive airway disease may mimic asthma, thereby contributing to the high rate of clinical diagnosis. Endocrine disorders are also prominent clinical features among patients with pediatric CVID including growth hormone and adrenal deficiencies, hypothyroidism, and ovarian and testicular failure (13, 14). The risk of malignancies in CVID increases with age; however, single pediatric cases of Hodgkin (15) and non-Hodgkin lymphoma (16) have also been reported. The marked heterogeneity of clinical symptomatology in pediatric patients with CVID reflects the complexity of the genetic background (17), immune response abnormalities (18), and the diversity of organ-specific immunopathology in this disease. The high variability of clinical features in affected children renders the early recognition of CVID challenging and requires high pediatricians' awareness and in-depth multidisciplinary diagnostics and monitoring (19).

The pathomechanisms of immune dysregulation with autoimmune, lymphoproliferative, and autoinflammatory phenotypes in pediatric CVID have not been hitherto clearly defined. Several components of the lymphocyte compartment, such as but not limited to increased immature CD21low B cell and decreased switched memory B-cell subsets along with depleted naive T CD4+ helper and T regulatory, as well as T CD8+ cytotoxic T cells, have been proposed as biomarkers (18, 20).

Herein, we sought to determine the frequency of immune dysregulation in the form of lymphoproliferative, autoimmune, and autoinflammatory disorders in children affected with CVID. To better define the diagnostic approach, we also investigated clinical and immunophenotypic prognostic markers of the immune dysregulation phenotypes in patients with pediatric CVID.

## Patients and Methods

We retrospectively reviewed a group of children diagnosed and treated for CVID in our tertiary referral center for pediatric immunology. In all the children studied, the definitive diagnosis of CVID had been established according to the current ESID Registry working definitions and guidelines for the clinical and immunodiagnostic recognition of CVID (7). In agreement with these criteria, an in-depth analysis of clinical symptomatology, including increased susceptibility to infections, autoimmune manifestations, granulomatous disease, organ-specific immunopathology, polyclonal lymphoproliferation, malignancy, and the contributory family history of a family member affected with antibody deficiency, was done. Secondary causes of hypogammaglobulinemia, such as malabsorption syndrome, nephrotic syndrome, malnutrition, malignancy, and immunosuppressive therapy, in particular with B-cell depleting medications, were excluded. The whole study group was then divided into an infection-only group and an immune dysregulation group for further comparative clinical and immunological evaluation.

In all the children studied, the individual patients' immunophenotype based on peripheral blood lymphocyte flow cytometric approach, in accordance with the methods and age-matched reference values described in source reports (21, 22), was determined. Detailed information on the protocol used for peripheral blood lymph cell preparation and flow cytometric immunophenotyping are available in the Supplementary Material. The analysis included absolute counts and relative values of the total lymphocyte population, total CD19+ B cells, and the following B-cell subsets: immature CD19+CD21lo, immature activated CD19+CD38loCD21lo, transitional CD19+CD38hisIgMhi, naive CD19+CD27-sIgD+, non-switched memory CD19+CD27+sIgD+, switched-memory CD19+CD27+IgD–, and CD19+CD38hisIgM– plasmablasts. Within the CD3+ T lymphocyte compartment, the distribution of CD3+CD4+ T helper (Th) and CD3+CD8+ T cytotoxic (Tc) was determined. Among Th cells, the following subsets were distinguished: CD3+CD4+ CD45RA+CD31+ recent thymic emigrants (RTE), CD3+CD4+CD27+CD45RA+ naive, CD3+CD4+CD27+CD45RO+ central memory, CD3+CD4+CD27–CD45RO+ effector memory, CD3+CD4+CD27–CD45RA+ terminally differentiated, CD3+CD4+ CD45RO+CD185+ (CXCR5+) follicular Th cells, and CD3+CD4+CD45RO+CD127–CD25++ T regulatory (Treg) cells. The analysis of Tc cells covered CD3+CD8+CD197+CD27+CD45RA+ naive, CD3+CD8+CD197+CD27+CD45RO+ central memory, CD3+CD8+CD197-CD27-CD45RO+ effector memory, and CD3+CD8+CD197-CD27-CD45RA+ terminally differentiated cells. The CD3-CD16+CD56+ natural killer (NK) cells were also identified.

Absolute counts and relative values of the analyzed lymphocyte subsets were compared between infection-only and immune dysregulation groups using Mann–Whitney U-test and Student's *t*-test in those cases when the distribution of values was normal. A significance cutoff value of *p* = 0.05 was used for each test. Differences in the distribution of out-of-range values were tested using Pearson's chi-square test and Fisher's exact test in those cases when the assumptions for the chi-square test were not met. Again, the statistical significance level was set for each test at *p* = 0.05. All analyses were performed with the use of Statistica v. 13.3.

## Results

The retrospectively reviewed group consisted of 39 children, 29 boys and 10 girls, aged from 4 to 18 years (mean age, 12.77 years or 12 9/12; median age, 14 years). The infection-only group consisted of seven patients and the immune dysregulation group consisted of 32 patients.

### Clinical Phenotypes

Whereas, recurrent sinopulmonary infections were present in all (100%) of the children studied, an unexpectedly high rate of non-infectious disorders and immune dysregulation phenotypes were observed in as many as 32 (82.05%) patients, compared with infection-only phenotypes limited to seven (17.95%) male patients. The most common inflammatory comorbidity was asthma, diagnosed in 21 (53.85%) patients. The second most frequent immune dysregulation group was autoimmune disorders, present in 18 (46.15%) of the children studied with a high rate of autoimmune endocrinopathies and predominating autoimmune thyroiditis in as many as 10 (25.64%) of the CVID-affected children. Lymphoproliferation was seen in 14 children (35.90%) and, among them, lymphadenopathy occurred in nine (23.08%), GLILD in seven (17.95%), hepatosplenomegaly in five (12.82%), and cutaneous granulomas in two (5.13%) cases. GLILD was diagnosed statistically significantly (*p* < 0.001) more often in CVID children with lymphadenopathy (in six of nine patients) and more often in children with autoimmunity, but this difference was not statistically significant. Finally, malignancies occurred in two female patients (5.13%): papillary thyroid cancer in the first one and T-cell lymphoblastic leukemia in the other one.

**Figure 1:**
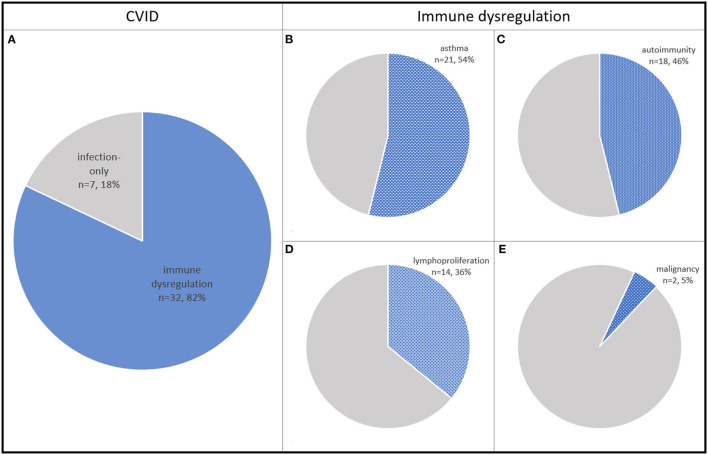
The proportions of CVID children with immune dysregulation features **(A)**, asthma **(B)**, autoimmunity **(C)**, lymphoproliferation **(D)**, and malignancy **(E)**.

**Figure 2:**
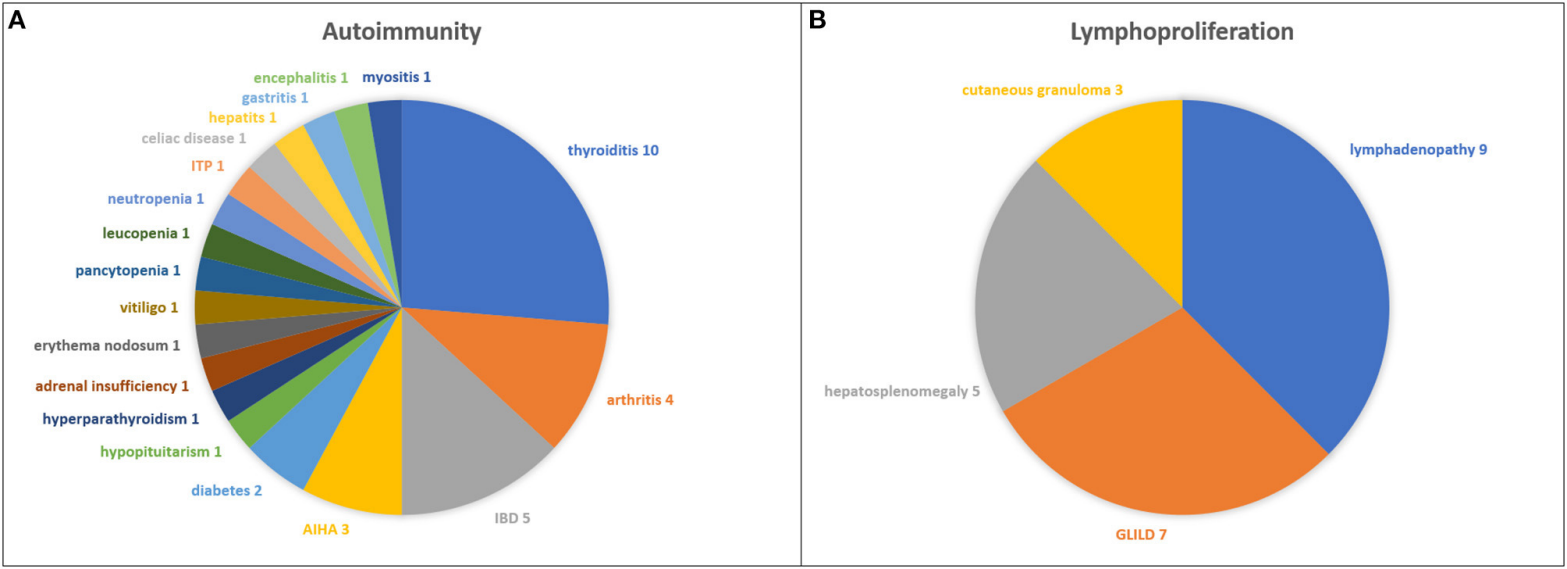
The spectrum of clinical diagnoses and the numbers of CVID children affected with autoimmune **(A)** and lymphoproliferative **(B)** disorders.

In Table 1, infectious and non-infectious clinical manifestations in all the CVID children studied have been summarized. The proportions of CVID children with immune dysregulation features, asthma, autoimmunity, lymphoproliferation, and malignancy are shown in Figure 1. The spectrum of clinical diagnoses and the numbers of CVID children affected with autoimmune and lymphoproliferative disorders are shown in Figure 2.

**Table 1:** Infectious and non-infectious complications in patients with pediatric CVID.

**Pt No**	**Sex**	**Age at onset**	**Age at report**	**Infections**	**Immune dysregulation**			
					**Autoinflammation**	**Autoimmunity**	**Lymphoproliferation**	**Malignancy**
1	M	2	4	Bronchitis Pneumonia		Arthritis		
2	F	2	4	Bronchitis Pneumonia	Asthma			
3	F	2	4	Bronchitis Enterocolitis	Asthma			
4	M	2	7	Bronchitis Pneumonia Gastroenteritis		Thyroiditis		
5	M	4	7	Bronchitis Urinary tract infections	Recurrent fevers			
6	M	4	7	Bronchitis Gastroenteritis	Asthma			
7	M	1	7	Bronchitis Pneumonia				
8	M	6	8	Sinusitis Bronchitis	Asthma			
9	M	4	8	Sinusitis Bronchitis	Asthma			
10	F	6	9	Sinusitis Bronchitis				Leukemia ALL-T
11	F	6	10	Bronchitis Recurrent labial herpes	Asthma	Thyroiditis ITP	GLILD	
12	M	6	10	Sinusitis Bronchitis	Asthma			
13	F	4	11	Sinusitis Bronchitis	Asthma	Thyroiditis AIN		
14	M	2	11	Bronchitis Pneumonia Enterocolitis				
15	M	6	12	Sinusitis Bronchitis	Asthma	Diabetes AIHA Leucopenia Thyroiditis	Cutaneous granuloma	
16	M	6	12	Sinusitis Bronchitis				
17	M	2	13	Sinusitis Bronchitis Pneumonia	Asthma	Arthritis Myositis Thyroiditis Hypopituitarism	Lymphadenopathy Hepatosplenomegaly GLILD Cutaneous granuloma	
18	M	2	13	Sinusitis Bronchitis		IBD Arthritis		
19	M	6	13	Sinusitis Bronchitis Parotitis	Asthma		Lymphadenopathy GLILD	
20	M	8	14	Sinusitis Bronchitis Enteritis	Asthma	Erythema nodosum	Lymphadenopathy	
21	M	10	14	Sinusitis Bronchitis		Diabetes Thyroiditis Adrenal insufficiency		
22	F	8	14	Sinusitis Bronchitis	Asthma		Cutaneous granuloma	
23	F	10	15	Sinusitis Bronchitis		Thyroiditis Vitiligo IBD		Papillary thyroid cancer
24	M	9	15	Sinusitis Bronchitis	Asthma			
25	M	6	15	Sinusitis Bronchitis		Hyperparathyroidism		
26	M	6	15	Sinusitis Bronchitis Pneumonia Bronchiestasis		Glomerulonephritis		
27	F	10	16	Sinusitis Bronchitis Urinary tract infections	Asthma			
28	M	12	16	Sinusitis Bronchitis		AIHA	Lymphadenopathy Hepatosplenomegaly	
29	M	13	16	Sinusitis Enterocolitis				
30	F	15	17	Sinusitis Pneumonia Myocarditis	Asthma	Celiac disease		
31	M	10	17	Sinusitis Bronchitis Meningitis				
32	M	4	17	Sinusitis Bronchitis Otitis		IBD Gastritis Encephalitis	Lymphadenopathy GLILD	
33	M	14	18	Otitis Sinusitis Bronchitis Bronchiectasis		Pancytopenia Thyroiditis Hepatitis IBD	Lymphadenopathy Hepatosplenomegaly GLILD	
34	M	16	18	Sinusitis Bronchitis Bronchiectasis			Lymphadenopathy Hepatosplenomegaly GLILD	
35	M	13	18	Sinusitis Bronchitis	Asthma			
36	M	14	18	Sinusitis Bronchitis Pneumonia Otitis	Asthma	Thyroiditis AIHA IBD	Lymphadenopathy Hepatosplenomegaly GLILD	
37	M	8	18	Sinusitis Bronchitis	Asthma			
38	F	12	18	Sinusitis Bronchitis	Asthma	Thyroiditis	Lymphadenopathy	
39	M	4	18	Sinusitis Bronchitis	Asthma	Arthritis	AIN	

### B and T Lymphocyte Compartments

**Figure 3:**
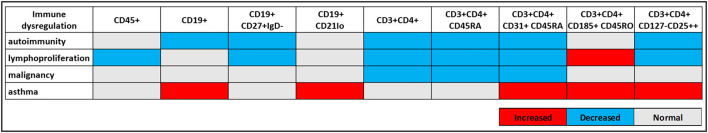
The distribution of out-of-range absolute counts of B and T lymph cell subsets, total CD45+ lymph cells, total CD19+ B cells, CD19+CD27+IgD– switched memory B cells, CD19+CD21lo immature B cells, CD3+CD4+ T helper cells, CD3+CD4+CD45RA naive T helper cells, CD3+CD4+CD31+CD45RA recent thymic emigrants, CD3+CD4+CD185+CD45RO follicular T helper cells, and CD3+CD4+CD127–CD25++ T regulatory cells, in immune dysregulation groups.

The detailed results of flow cytometric analysis of B, Th, Tc, and NK cells in all patients with CVID have been displayed in Supplementary Tables 1, 2A,B, 3, respectively. The distribution of out-of-range numbers of B and T lymph cell subsets in particular immune dysregulation groups is shown in Figure 3. The analysis of absolute counts and relative values of the total lymphocyte population, total B, T, and NK cell compartments, and subpopulations did not show statistically significant differences between infection-only and immune dysregulation groups of the CVID children studied. However, the lymphocyte absolute counts were lower in the latter group than in patients with infection-only CVID. The further analysis of differences in the distribution of out-of-range absolute counts and relative values of lymph cell subsets revealed comparable percentages of incorrect results in infection-only and immune dysregulation groups. Of note, low relative numbers of CD19+ B lymph cells were exceptional, showing a striking predilection and statistical significance (*p* = 0.028) in male patients with infection-only CVID (28.57% vs. 0% in immune dysregulation groups).

Within the immune dysregulation group, we sought to determine whether in children with distinct CVID phenotypes, such as CVID and autoimmunity, CVID and lymphoproliferation, CVID and asthma, and, finally, CVID with malignancy, the frequencies of abnormal absolute or relative numbers of the analyzed lymph cell subsets show differences in comparison to CVID children without those complications.

In children with CVID and concomitant autoimmune disorders, absolute numbers of switched memory B cells, RTE, and Treg cells and their relative numbers were statistically significantly lower than in the group without autoimmunity (*p* = 0.047, *p* = 0.026, *p* = 0.016, and *p* = 0.041, respectively). In the CVID with autoimmunity group, absolute counts of total CD19+ B cells, total CD4+ Th cells, and naive CD4+ Th cells were lower than in the group without autoimmunity, but this difference was not significant. The analysis of differences in the distribution of out-of-range values showed that, in the group of children with CVID and autoimmunity, decreased relative and absolute numbers of Treg cells occurred more often than in the group without autoimmunity (61.11% vs. 33.33% and 72.22 vs. 23.81%, respectively), and, for absolute Treg counts, this difference was statistically significant (*p* = 0.003).

In the CVID and lymphoproliferation group, absolute counts of lymphocytes, absolute counts and relative values of switched memory B cells and naive CD4+ T helper cells, and absolute counts of RTE were statistically significantly lower than in the group without lymphoproliferation (*p* = 0.018, *p* = 0.024, *p* = 0.023, *p* = 0.024, *p* = 0.034, and *p* = 0.043, respectively). The absolute count of CD3+CD4+CD127–CD25++ Treg cells was also lower in CVID children with lymphoproliferation, but this difference was not statistically significant. The CVID with lymphoproliferation group was also characterized by statistically significantly higher percentages of out-of-range absolute Treg cell numbers (71.43% vs. 32.00% and *p* = 0.018).

We also applied the Mann–Whitney U-test to compare the absolute counts and relative values in two CVID children who developed malignant disorders and in CVID children without malignancies. However, because of the very small numbers of patients in the first group, interpretation of statistical data is significantly limited and its predictive value may be debated. In the two female patients with malignancies, the absolute counts of CD4+ T helper cells were statistically significantly lower than in the CVID without malignancy group (*p* = 0.044), and the numbers of T CD4+ naive cells and RTE were in the malignancy group lower but not statistically significantly. The analysis of the distribution of out-of-range values in naive Th and RTE cells showed that they were noted statistically significantly more frequently in the malignancy group (100% vs. 8.11%, *p* = 0.014, and 100% vs. 10.81%, *p* = 0.02, respectively).

Finally, median numbers and relative values of lymph cell subsets were analyzed in the CVID and asthma group and compared with patients with CVID without asthma. Surprisingly, in asthmatic children with CVID, the absolute counts and percentage values of CD19+, and CD19+CD21lo B cells, RTE, Treg cells, and CXCR5+ Tfh cells were increased, but only relative values of the latter T-cell subset showed statistical significance (*p* = 0.040). In the CVID group without asthma, the distribution of lower absolute numbers of CD4+ T cells (27.78%) and absolute numbers and relative values of RTE cells (both 22.22%) was noted significantly more frequent than in the CVID with asthma group (all values within the normal range, *p* = 0.015, *p* = 0.037, and *p* = 0.037, respectively).

Figures 4.1–4.4 display the results of the statistical analysis including correlations between out-of-range absolute counts and relative values of B lymph cell subsets and immune dysregulation features (**A–D**). The results of the statistical analysis of T lymph cell subsets in immune dysregulation are displayed in Figures 5.1–5.4 (**A–F**).

**Figure 4:**
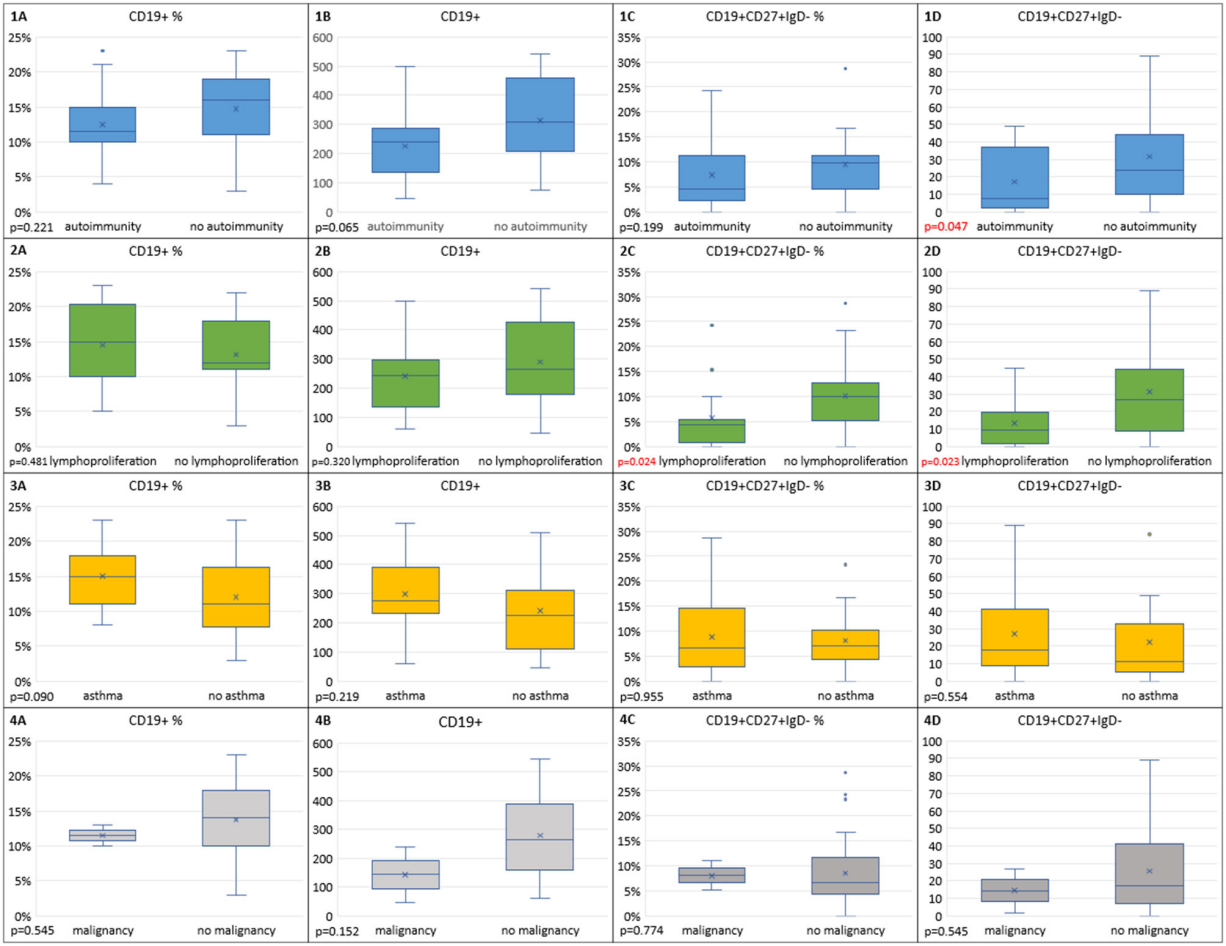
Box plots reflecting distributions of out-of-range relative values **(A,C)** and absolute counts **(B,D)** of total B cells and switched memory B cells in immune dysregulation vs. no dysregulation groups. **(1)** Autoimmunity vs. no autoimmunity (*n* = 18 vs. *n* = 21, respectively); **(2)** Lymphoproliferation vs. no lymphoproliferation (*n* = 14 vs. *n* = 25); **(3)** Asthma vs. no asthma (*n* = 21 vs. *n* = 18); and **(4)** Malignancy vs. no malignancy (*n* = 2 vs. *n* = 37).

**Figure 5:**
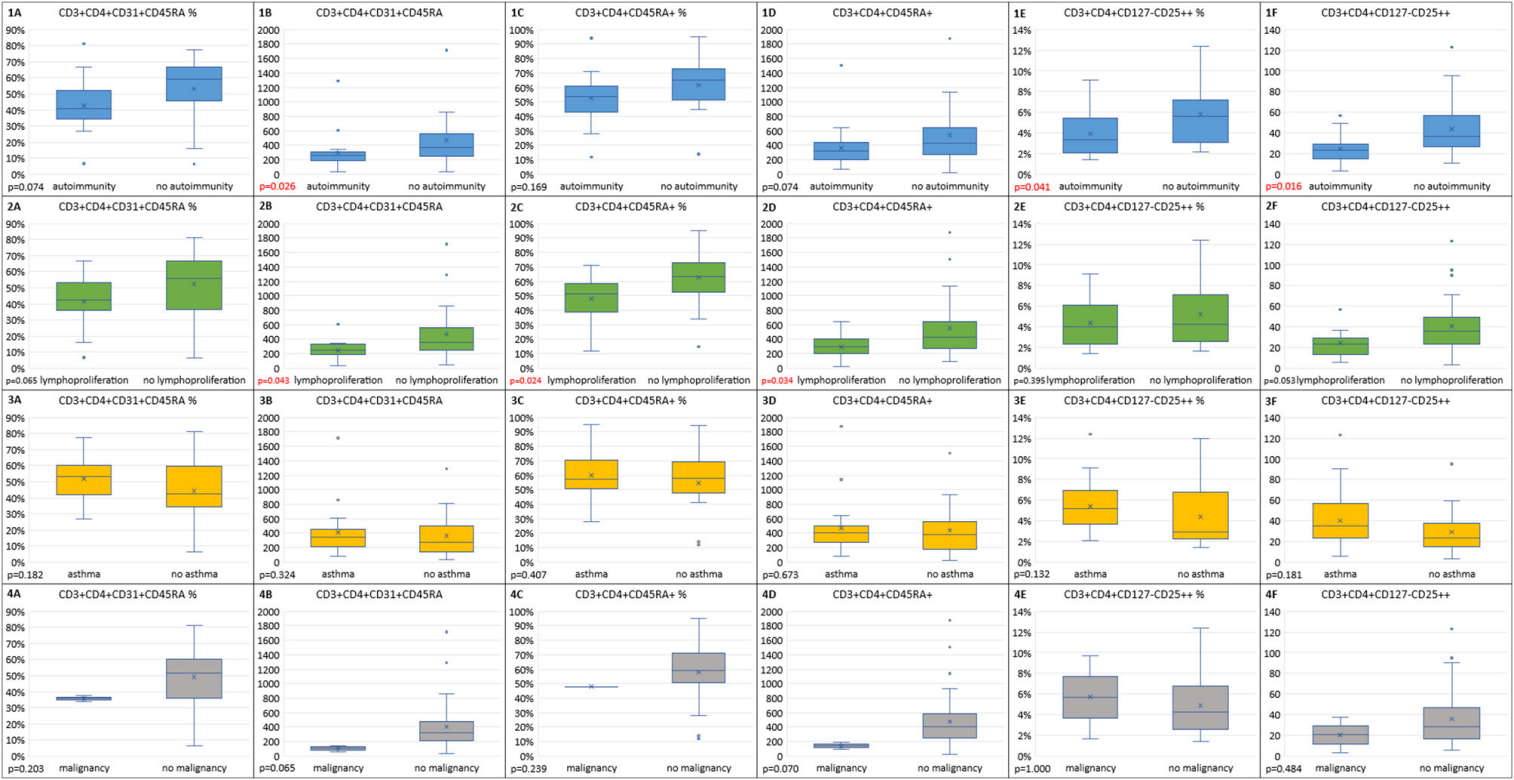
Box plots reflecting distributions of out-of-range relative values **(A,C,E)** and absolute counts **(B,D,F)** of RTE, naive T helper, and regulatory Th cells in immune dysregulation vs. no dysregulation groups. **(1)** Autoimmunity vs. no autoimmunity (*n* = 18 vs. *n* = 21, respectively); **(2)** Lymphoproliferation vs. no lymphoproliferation (*n* = 14 vs. *n* = 25); **(3)** Asthma vs. no asthma (*n* = 21 vs. *n* = 18); and **(4)** Malignancy vs. no malignancy (*n* = 2 vs. *n* = 37).

## Discussion

Despite the low prevalence of CVID in the pediatric population, the individual burden of this primary immunodeficiency on affected children is remarkable. The pediatric CVID is a notorious disease for its unfavorable outcomes and the spectrum of complications from infections through autoimmune disorders, lymphoproliferative diseases, autoinflammatory processes, to malignancy. The analysis of CVID-related comorbidities in our pediatric patients shows that infections and infectious complications are admittedly the most frequently recognized manifestations of CVID (23, 24), as seen in our group in 100% of patients; nonetheless, immune dysregulation is a spectrum of illness remarkably affecting our pediatric CVID studied, similarly to other reports (1, 25–27).

In our cohort, the most frequently recognized non-infectious complication was asthma, diagnosed in as many as 54% of patients. This belongs to the highest hitherto reported rates of asthma in pediatric CVID, where incidence has been estimated to range between 9–15% (2) and 50–57% (28, 29). Nevertheless, it must be highlighted that neither atopic comorbidities nor elevated total immunoglobulin E (IgE) and allergen-specific IgE have been observed in any of the children studied. This observation is consistent with the results of a large multicenter cohort of patients with CVID in whom low serum IgE proved to be a sensitive and specific marker of the humoral immunodeficiency disease albeit did not allow for detecting allergen sensitization (30). Furthermore, asthma had been diagnosed in our patients with CVID before the diagnosis of CVID has been established, indicating possible misdiagnoses of asthma in infection-induced and immunodeficiency-related obstructive airway disease in some cases. One may also assume that not allergic dysregulation but an autoinflammatory skewed immune response accompanied by increased numbers of immature CD19+CD21lo B cells and naive Th and Treg cells is distinctive for the CVID phenotype in those children. In a systematic review of all existing CVID cases in databases (31), including pediatric cohort, the prevalence of asthma at the initial presentation of CVID reached 31% of affected individuals. Likewise, IgE-dependent hypersensitivity to environmental allergens has been documented; thereby, atopic background has not been proven in all reported patients, all the more supporting the hypothesis of possible ascribing the immunodeficiency-related obstructive airway disease to atopic asthma. Finally, because of the disturbed immunosurveillance, the clinical symptomatology of both conditions may overlap significantly and therefore, those children diagnosed with asthma who do not show a satisfactory response to standard therapy should be considered as potential candidates for CVID.

The second most frequent immune dysregulation in our children with CVID was autoimmune disorders, occurring in as many as 46% of patients and comprising a wide spectrum of clinical conditions. An exceptionally high rate of endocrine disorders has been noted in our CVID cohort, with autoimmune thyroiditis as the most prominent clinical feature, diagnosed in 10 of 18 children with CVID and autoimmunity. Other autoimmune endocrinopathies included diabetes in two patients, as well as adrenocortical insufficiency, hypopituitarism, and hyperparathyroidism, all of these conditions seen in individual cases. Our findings are consistent with previous studies that demonstrated the presence of autoimmune hormonal dysfunctions, including hypothyroidism, hypogonadism, and adrenal insufficiency in adult patients with CVID (13). In pediatric patients presenting with CVID and adrenocorticotropin deficiency, the suspicion of DAVID syndrome (deficient anterior pituitary with variable immune deficiency) due to pathogenic variants in the *NF-*κ*B2* (nuclear factor kappa B2) gene should be raised (32, 33). Other autoimmune disorders included cytopenias, most frequently AIHA in three of 18 patients with CVID and autoimmunity, and autoimmune thrombocytopenic purpura (ITP), autoimmune neutropenia (AIN), leucopenia, and pancytopenia, which were present in single cases. Our findings, showing a remarkable association between autoimmune cytopenias and CVID in the children studied, with their prevalence in five of total 39 patients and of 18 patients with autoimmunity (12.82 and 27.77%, respectively), are supported by other reports indicating a link between immune-mediated disturbances within hematopoietic cell lineages (34, 35). Other autoimmune phenomena demonstrated by our pediatric patients concurrently with CVID and expanding the broad clinical phenotype were rheumatologic diseases (e.g., arthritis, as observed in four patients, and myositis), gastrointestinal disorders [that is inflammatory bowel disease (IBD) in as many as five patients] autoimmune hepatitis, gastritis, and celiac disease, as well as cutaneous manifestations, such as erythema nodosum and vitiligo. Autoimmune cytopenias coexisting with clinical features of organ-specific or systemic immunodysregulatory phenotype in pediatric patients with CVID and genetically defined IEIs, such as STAT3 GOF (signal transducer and activator of transcription 3 gain-of-function) disease, APDS [activated PI3Kδ (phosphoinositide-3-kinase delta) syndrome], FAS-ALPS (autoimmune lymphoproliferative syndrome), and Kabuki syndrome, have also been recently reported (36). Noteworthy, we have also found a link between autoimmune complications and distinctive lymphocyte immunophenotype with decreased numbers of total CD19+ and CD19+CD27+IgD– switched memory B cells and, within the T-cell compartment, decreased numbers of total CD4+Th, naive CD4+CD45RA+ Th cells, RTE, and CD25++ Treg cells.

**Figure 6:**
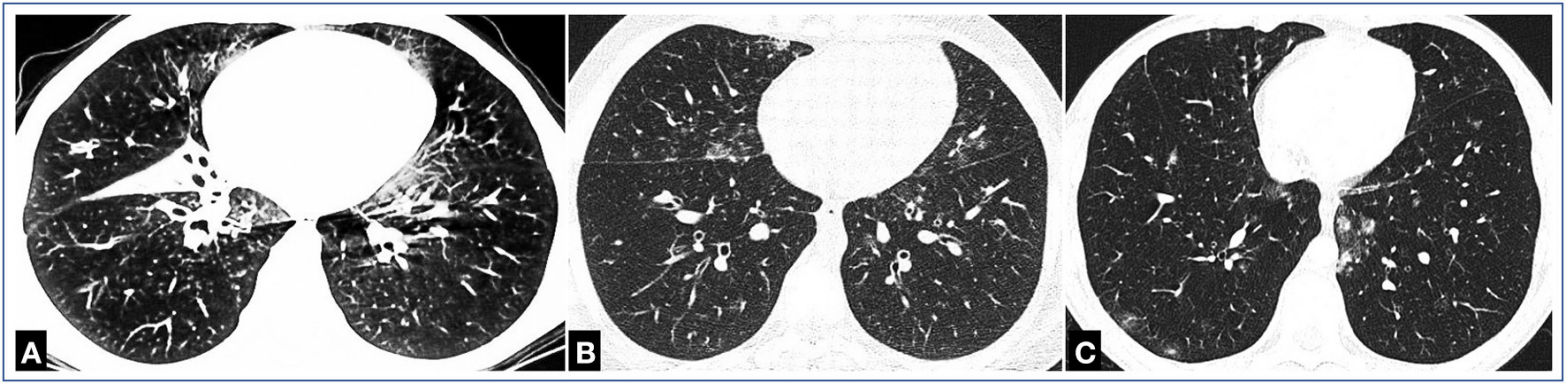
Thoracic computed tomography (CT) imaging of patients with CVID with granulomatous lymphocytic interstitial lung disease (GLILD). **(A)** Multiple confluent nodules, tree-in-bud pattern reflecting bronchiolitis, interstitial pneumonia, peribronchial thickening and bronchiectasis, atelectasis, and fibrosis of the right middle lobe. **(B)** Patchy, confluent ground-glass opacities, tree-in-bud pattern, single nodules. **(C)** Multiple, irregular, disseminated perivascular and subpleural nodules, ground-glass opacities.

Although numerous heterogeneous disturbances of immune homeostasis within the peripheral lymphocyte pools have been observed in CVID (20, 37–39), yet similar lymphocyte subset abnormalities in pediatric CVID have also been reported (18). Furthermore, a flow cytometric analysis of peripheral B and T lymph cells in children manifesting lymphoproliferative disorders has revealed developmental abnormalities of total CD19+ and switched memory B cells along with T cells subsets, such as RTE, naive Th, and Treg cells. These flow cytometric features of dysfunctional B and T lymph cell immune response were accompanied by increased numbers of follicular Th (fTh) cells, which not only may be considered as diagnostic biomarkers of costimulation to B cells and their impairment may point to CVID in children (40), but they have also been proposed to trigger autoreactive B-cell populations, thereby contributing to the development of autoimmune and lymphoproliferative complications in CVID (37, 41). In our study group showing lymphoproliferative disorders, the clinical diagnoses included lymphadenopathy, GLILD (as shown in Figure 6), hepatosplenomegaly, and cutaneous granulomas. It is worth noting that, in all our patients with CVID presenting with GLILD, other extrapulmonary, systemic features of immune dysregulation have been observed, in the form of lymphadenopathy, granulomatous liver and spleen disease, and cutaneous granulomatosis, supporting the hypothesis of the generalized nature of the granulomatous disease in CVID (9, 10, 42, 43). It is also worth pointing out that, in our patients with pediatric CVID, same as in children with autoimmune cytopenias reported in (36), either GLILD or any other granulomatous lymphoproliferative manifestations were not associated with increased numbers of immature B-cell subset with CD19+CD21lo immunophenotype, in which expansion has been hypothesized to be a predictor of granulomatous complications in CVID (8, 44, 45).

Finally, malignant disorders have been the least frequent immune dysregulation sequelae in our pediatric CVID cohort, affecting two patients: one with thyroid cancer and the other one with T-cell lymphoblastic leukemia that constitutes 5.12% among all the 39 CVID children studied and 6.25% among 32 children with CVID and immune dysregulation. The rate of malignancies has proved to be even higher than the overall risk of malignancy in IEI that has been estimated to range from 4.7 to 5.7% (46) and reach 20% in adulthood (47), with non-Hodgkin lymphoma as the most frequent malignancy in patients with pediatric CVID (16, 30, 47). The susceptibility to malignant transformation in CVID has been linked to the concept of impaired immunosurveillance, predisposition to lymphoproliferative disorders, ineffective clearance of oncogenic viruses, as well as genetic and epigenetic background (48–51). In our patients with CVID and malignancy, similarly as in patients with autoimmunity and lymphoproliferation, we have demonstrated decreased numbers of RTE, total Th, and fTh cells, while, in contrast to other patients with CVID and immune dysregulation, we have not observed remarkable disturbances within the B-cell pools and Treg cells.

## Conclusion

The pediatric CVID is characterized by a remarkably high incidence of immune dysregulation sequelae involving autoinflammatory, autoimmune, lymphoproliferative, and malignant disorders. Although infections and infectious complications are the most frequent manifestations in children with CVID, the disease has no longer been perceived as infection-only presenting immunodeficiency, thereby underscoring heterogeneity of its clinical phenotypes. The multiplicity of systemic and organ-specific immunopathologies poses the need for increasing physicians' awareness and providing care by a multidisciplinary team to affected pediatric patients.

Although an in-depth flow cytometric analysis of children presenting with CVID and immune dysregulation points to developmental disturbances within the T helper cell pool, one may assume that, in this group of children, CVID shows features of a combined immunodeficiency with predominating antibody production defect rather than an isolated intrinsic B-cell dysfunction. The flow cytometric approach may therefore facilitate identification of patients with pediatric CVID showing particular vulnerability to immune dysregulation. Further research studies are required to explicitly define mechanisms related to mutual interactions between B and T lymph cells, thus predisposing to immune dysregulation features in pediatric CVID.

## Data Availability Statement

The raw data supporting the conclusions of this article will be made available by the authors, without undue reservation.

## Ethics Statement

Ethical review and approval was not required for the study on human participants in accordance with the local legislation and institutional requirements. Written informed consent to participate in this study was provided by the participants' legal guardian/next of kin.

## Author Contributions

AS-P was responsible for the conception and design of the study, the acquisition and interpretation of data, and drafting the manuscript. KT-J contributed to the design of the study, the acquisition and interpretation of data, and drafting the manuscript. ES and NP helped in the acquisition and analysis of data and drafting the manuscript. All authors contributed to the article and approved its final version.

## Conflict of Interest

The authors declare that the research was conducted in the absence of any commercial or financial relationships that could be construed as a potential conflict of interest.

## Publisher's Note

All claims expressed in this article are solely those of the authors and do not necessarily represent those of their affiliated organizations, or those of the publisher, the editors and the reviewers. Any product that may be evaluated in this article, or claim that may be made by its manufacturer, is not guaranteed or endorsed by the publisher.
