# Supplementary material for: Immune Dysregulation in Pediatric Common Variable Immunodeficiency: Implications for the Diagnostic Approach
Source: Front Pediatr. 2022 Mar 23;10:855200. doi: 10.3389/fped.2022.855200 (PMC8983883; doi:10.3389/fped.2022.855200)
Supplement: Supplementary file 1 [file Table_1.DOCX]

Supplemental Table 1. The flow cytometric analysis of B cell subpopulations in CVID children

| **Patient No** | **B cell subpopulations** | | | | | | | | | | | | | | | | | |
| --- | --- | --- | --- | --- | --- | --- | --- | --- | --- | --- | --- | --- | --- | --- | --- | --- | --- | --- |
|  | **CD45** | | **CD19** | | **CD19+**  **CD38hi sIgMhi** | | **CD19+**  **CD27-sIgD+** | | **CD19+**  **CD27+sIgD+** | | **CD19+**  **CD27+sIgD-** | | **CD19+**  **CD38hi sIgM-** | | **CD19+**  **CD21lo** | | **CD19+**  **CD38lo CD21lo** | |
| 1 | 39% | 3861 | 11% | 425 | 5.3% | 23 | 76.2% | 324 | 8.8% | 37 | 11.6% | 49 | 0.5% | 2 | 7.4% | 31 | 7.4% | 31 |
| 2 | 52% | 5862 | 8% | 464 | 3.5% | 16 | 76.1% | 353 | 7.7% | 36 | 12.7% | 59 | 0.0% | 0 | 3.5% | 16 | 3.5% | 16 |
| 3 | 27% | 1340 | 18% | 243 | 12.9% | 31 | 90.5% | 220 | 3.8% | 9 | 3.0% | 7 | 0.4% | 1 | 10.1% | 25 | 4.7% | 11 |
| 4 | 28% | 3865 | 4% | 178 | 1.6% | 3 | 64.4% | 115 | 12.3% | 22 | 23.3% | 42 | 0.0% | 0 | 4.9% | 9 | 4.9% | 9 |
| 5 | 39% | 2317 | 21% | 498 | 10.1% | 50 | 69.2% | 344 | 11.8% | 59 | 16.8% | 84 | 0.2% | 1 | 4.2% | 21 | 2.9% | 14 |
| 6 | 33% | 2714 | 20% | 543 | 8.7% | 47 | 77.9% | 423 | 8.1% | 44 | 9.9% | 54 | 1.1% | 6 | 8.7% | 47 | 3.8% | 21 |
| 7 | 43% | 2338 | 19% | 437 | 8.9% | 39 | 76.6% | 335 | 9.5% | 42 | 10.0% | 44 | 0.2% | 1 | 9.1% | 40 | 3.2% | 14 |
| 8 | 30% | 1769 | 16% | 313 | 13.6% | 43 | 81.4% | 255 | 4.3% | 13 | 11.2% | 35 | 0.8% | 3 | 3.8% | 12 | 1.9% | 6 |
| 9 | 44% | 2210 | 14% | 310 | 0.2% | 1 | 52.4% | 162 | 14.3% | 44 | 28.6% | 89 | 0.0% | 0 | 28.2% | 87 | 8.3% | 26 |
| 10 | 31% | 1835 | 13% | 241 | 11.8% | 28 | 76.4% | 184 | 11.8% | 28 | 11.0% | 27 | 1.1% | 3 | 16.8% | 40 | 11.1% | 27 |
| 11 | 37% | 2128 | 23% | 499 | 12.8% | 64 | 83.4% | 416 | 8.3% | 41 | 4.4% | 22 | 2.0% | 10 | 9.5% | 47 | 6.4% | 32 |
| 12 | 33% | 1248 | 9% | 119 | 7.2% | 9 | 65.2% | 77 | 13.5% | 16 | 15.0% | 18 | 0.4% | 0 | 16.9% | 20 | 15.7% | 19 |
| 13 | 55% | 1745 | 16% | 292 | 17.1% | 50 | 77.9% | 227 | 2.6% | 8 | 15.4% | 45 | 2.9% | 8 | 15.5% | 45 | 10.3% | 30 |
| 14 | 45% | 2786 | 11% | 315 | 8.1% | 25 | 70.9% | 223 | 17.5% | 55 | 10.4% | 33 | 1.4% | 4 | 16.1% | 51 | 2.4% | 8 |
| 15 | 36% | 1626 | 15% | 252 | 13.4% | 34 | 91.3% | 230 | 3.3% | 8 | 4.9% | 12 | 1.4% | 4 | 3.8% | 10 | 1.7% | 4 |
| 16 | 29% | 2873 | 3% | 99 | 13.0% | 13 | 89.3% | 88 | 2.7% | 3 | 8.0% | 8 | 1.4% | 1 | 10.1% | 10 | 1.4% | 1 |
| 17 | 34% | 1412 | 8% | 127 | 20.7% | 26 | 95.4% | 121 | 2.3% | 3 | 0.0% | 0 | 2.6% | 3 | 5.7% | 7 | 2.6% | 3 |
| 18 | 23% | 1559 | 12% | 201 | 9.6% | 19 | 79.3% | 160 | 15.0% | 30 | 4.3% | 9 | 0.0% | 0 | 5.3% | 11 | 4.3% | 9 |
| 19 | 37% | 2070 | 21% | 460 | 5.1% | 23 | 91.1% | 419 | 4.6% | 21 | 2.1% | 10 | 0.0% | 0 | 17.2% | 79 | 4.0% | 18 |
| 20 | 32% | 1626 | 21% | 364 | 31.2% | 114 | 98.5% | 359 | 0.8% | 3 | 0.2% | 1 | 0.2% | 1 | 6.8% | 25 | 1.3% | 5 |
| 21 | 32% | 1236 | 20% | 247 | 11.4% | 28 | 87.4% | 216 | 7.5% | 19 | 3.0% | 7 | 1.2% | 3 | 5.4% | 13 | 4.2% | 10 |
| 22 | 26% | 1451 | 18% | 275 | 4.0% | 11 | 84.0% | 231 | 9.8% | 27 | 4.5% | 12 | 0.3% | 1 | 3.4% | 9 | 3.4% | 9 |
| 23 | 11% | 410 | 10% | 46 | 6.1% | 3 | 85.7% | 39 | 9.1% | 4 | 5.2% | 2 | 4.1% | 2 | 6.1% | 3 | 10.2% | 5 |
| 24 | 41% | 2289 | 11% | 258 | 4.0% | 10 | 81.0% | 209 | 6.6% | 17 | 9.3% | 24 | 0.1% | 0 | 7.0% | 18 | 5.1% | 13 |
| 25 | 35% | 1949 | 14% | 276 | 6.3% | 23 | 86.3% | 317 | 6.0% | 22 | 1.9% | 5 | 1.8% | 7 | 10.9% | 30 | 4.2% | 15 |
| 26 | 19% | 1710 | 4% | 74 | 10.3% | 8 | 91.7% | 68 | 5.6% | 4 | 0.0% | 0 | 0.0% | 0 | 7.7% | 6 | 5.1% | 4 |
| 27 | 27% | 1111 | 11% | 129 | 10.1% | 13 | 82.9% | 107 | 8.8% | 11 | 6.7% | 9 | 1.1% | 1 | 4.7% | 6 | 4.0% | 5 |
| 28 | 32% | 1231 | 5% | 75 | 2.3% | 2 | 83.2% | 63 | 8.8% | 7 | 5.6% | 4 | 1.1% | 1 | 8.0% | 6 | 5.7% | 4 |
| 29 | 39% | 2835 | 17% | 510 | 7.5% | 38 | 83.3% | 425 | 6.8% | 35 | 6.3% | 32 | 0.0% | 0 | 15.2% | 78 | 9.4% | 48 |
| 30 | 44% | 2156 | 12% | 264 | 5.4% | 14 | 58.9% | 155 | 22.6% | 60 | 15.7% | 41 | 0.5% | 1 | 8.3% | 22 | 6.5% | 17 |
| 31 | 26% | 1239 | 11% | 142 | 2.9% | 4 | 68.3% | 97 | 20.9% | 30 | 10.0% | 14 | 0.4% | 1 | 5.0% | 7 | 4.2% | 6 |
| 32 | 52% | 2807 | 11% | 299 | 7.4% | 22 | 76.9% | 230 | 7.1% | 21 | 10.1% | 30 | 1.4% | 4 | 12.2% | 37 | 10.5% | 31 |
| 33 | 31% | 971 | 7% | 71 | 3.1% | 4 | 72.8% | 52 | 18.5% | 13 | 4.3% | 3 | 0.0% | 0 | 4.3% | 3 | 7.1% | 9 |
| 34 | 18% | 864 | 23% | 207 | 23.5% | 49 | 87.0% | 180 | 7.0% | 14 | 4.3% | 9 | 1.0% | 2 | 13.2% | 27 | 9.2% | 19 |
| 35 | 22% | 2339 | 22% | 536 | 1.0% | 5 | 75.7% | 406 | 5.2% | 28 | 14.7% | 79 | 3.8% | 20 | 8.8% | 47 | 7.5% | 40 |
| 36 | 22% | 546 | 10% | 60 | 25.6% | 15 | 96.3% | 58 | 2.8% | 2 | 0.0% | 0 | 4.7% | 3 | 13.2% | 8 | 3.9% | 2 |
| 37 | 34% | 2074 | 18% | 389 | 6.5% | 25 | 86.9% | 338 | 7.2% | 28 | 4.4% | 17 | 1.9% | 7 | 12.6% | 49 | 8.4% | 33 |
| 38 | 31% | 1494 | 15% | 233 | 9.4% | 22 | 91.1% | 213 | 7.6% | 18 | 0.5% | 1 | 0.5% | 1 | 9.8% | 23 | 7.2% | 17 |
| 39 | 28% | 1551 | 10% | 160 | 10.3% | 16 | 54.3% | 87 | 12.7% | 20 | 24.3% | 39 | 1.6% | 3 | 17.3% | 28 | 13.0% | 21 |
