# Supplementary material for: Immune Dysregulation in Pediatric Common Variable Immunodeficiency: Implications for the Diagnostic Approach
Source: Front Pediatr. 2022 Mar 23;10:855200. doi: 10.3389/fped.2022.855200 (PMC8983883; doi:10.3389/fped.2022.855200)
Supplement: Supplementary file 2 [file Table_2.DOCX]

Supplemental Table 2a. Flow cytometric analysis of Th cell subpopulations in CVID children

| **Patient No** | **Th cell subpopulations** | | | | | | | | | | | | | | | | | | | |
| --- | --- | --- | --- | --- | --- | --- | --- | --- | --- | --- | --- | --- | --- | --- | --- | --- | --- | --- | --- | --- |
|  | **CD45** | | **CD3+** | | **CD3+CD4+** | | **CD3+CD4+**  **CD31+CD45RA+** | | **CD3+CD4+**  **CD27+CD45RA+** | | **CD3+CD4+**  **CD27+CD45RA-** | | **CD3+CD4+**  **CD27-CD45RA-** | | **CD3+CD4+**  **CD27-CD45RA+** | | **CD3+CD4+**  **CD185+CD45RO+** | | **CD3+CD4+**  **CD127-CD25++** | |
| 1 | 39% | 3861 | 67% | 2618 | 41% | 1596 | 81.1% | 1294 | 94.2% | 1504 | 3.3% | 52 | 0.8% | 13 | 0.6% | 10 | 9.1% | 7 | 1.7% | 27 |
| 2 | 52% | 5862 | 75% | 4475 | 38% | 2279 | 75.3% | 1717 | 82.2% | 1874 | 21.8% | 496 | 0.3% | 6 | 0.8% | 19 | 28.0% | 94 | 4.0% | 90 |
| 3 | 27% | 1340 | 65% | 892 | 42% | 582 | 77.6% | 451 | 85.7% | 499 | 22.5% | 131 | 0.5% | 3 | 0.4% | 3 | 25.4% | 22 | 4.6% | 27 |
| 4 | 28% | 3865 | 47% | 1905 | 18% | 722 | 33.8% | 244 | 61.6% | 445 | 45.3% | 327 | 5.9% | 42 | 1.0% | 7 | 11.2% | 29 | 6.8% | 49 |
| 5 | 39% | 2317 | 65% | 1583 | 33% | 811 | 67.5% | 548 | 72.7% | 590 | 25.6% | 207 | 2.4% | 20 | 0.1% | 1 | 17.5% | 38 | 7.2% | 59 |
| 6 | 33% | 2714 | 68% | 1852 | 44% | 1202 | 71.3% | 857 | 94.9% | 1141 | 3.3% | 40 | 1.0% | 12 | 1.7% | 21 | 28.3% | 38 | 3.3% | 39 |
| 7 | 43% | 2338 | 69% | 1636 | 42% | 981 | 69.2% | 697 | 78.1% | 766 | 21.1% | 207 | 0.7% | 7 | 1.0% | 10 | 26.2% | 61 | 2.4% | 23 |
| 8 | 30% | 1769 | 68% | 1324 | 31% | 598 | 60.4% | 361 | 69.3% | 414 | 43.8% | 262 | 1.7% | 10 | 0.0% | 0 | 31.0% | 54 | 5.9% | 35 |
| 9 | 44% | 2210 | 75% | 1668 | 37% | 813 | 59.3% | 482 | 71.2% | 579 | 42.7% | 347 | 2.1% | 17 | 0.0% | 0 | 24.9% | 57 | 7.0% | 57 |
| 10 | 31% | 1835 | 64% | 1186 | 21% | 389 | 37.7% | 147 | 48.0% | 187 | 57.2% | 223 | 4.5% | 18 | 0.8% | 3 | 19.5% | 63 | 9.7% | 38 |
| 11 | 37% | 2128 | 68% | 1476 | 42% | 912 | 66.6% | 607 | 71.0% | 647 | 22.7% | 207 | 2.9% | 26 | 2.6% | 24 | 40.7% | 113 | 6.3% | 57 |
| 12 | 33% | 1248 | 65% | 869 | 39% | 521 | 63.9% | 332 | 70.1% | 365 | 31.1% | 162 | 2.3% | 12 | 0.3% | 1 | 28.2% | 38 | 7.1% | 37 |
| 13 | 55% | 1745 | 68% | 1243 | 31% | 564 | 52.6% | 296 | 57.3% | 323 | 34.4% | 194 | 6.2% | 35 | 2.5% | 14 | 20.9% | 48 | 5.2% | 30 |
| 14 | 45% | 2786 | 64% | 1871 | 44% | 1273 | 59.4% | 757 | 63.1% | 804 | 40.2% | 512 | 1.4% | 18 | 0.6% | 7 | 34.7% | 138 | 3.0% | 38 |
| 15 | 36% | 1626 | 67% | 1122 | 37% | 616 | 56.2% | 346 | 70.6% | 435 | 33.1% | 204 | 5.1% | 31 | 0.2% | 1 | 16.5% | 25 | 3.7% | 23 |
| 16 | 29% | 2873 | 72% | 2096 | 27% | 794 | 6.3% | 50 | 14.7% | 117 | 93.5% | 742 | 4.5% | 36 | 0.4% | 3 | 12.8% | 89 | 12.0% | 95 |
| 17 | 34% | 1412 | 58% | 907 | 28% | 443 | 41.7% | 184 | 51.7% | 229 | 5.7% | 25 | 0.8% | 4 | 3.7% | 16 | 18.7% | 38 | 2.1% | 9 |
| 18 | 23% | 1559 | 78% | 1284 | 40% | 667 | 44.3% | 296 | 53.5% | 357 | 38.9% | 259 | 13.0% | 87 | 2.8% | 18 | 10.4% | 29 | 2.2% | 15 |
| 19 | 37% | 2070 | 66% | 1473 | 28% | 635 | 54.8% | 348 | 51.1% | 324 | 42.1% | 267 | 3.3% | 21 | 0.7% | 5 | 37.8% | 115 | 4.3% | 28 |
| 20 | 32% | 1626 | 62% | 1058 | 26% | 435 | 42.8% | 186 | 47.6% | 207 | 47.5% | 207 | 11.9% | 52 | 0.8% | 3 | 21.7% | 45 | 5.5% | 24 |
| 21 | 32% | 1236 | 57% | 711 | 33% | 407 | 36.0% | 147 | 41.5% | 169 | 54.0% | 220 | 6.3% | 25 | 0.2% | 1 | 29.0% | 65 | 4.3% | 18 |
| 22 | 26% | 1451 | 72% | 1117 | 39% | 605 | 35.8% | 216 | 44.7% | 270 | 55.7% | 337 | 5.1% | 31 | 2.6% | 16 | 17.9% | 66 | 2.2% | 14 |
| 23 | 11% | 410 | 75% | 344 | 42% | 190 | 33.9% | 64 | 47.8% | 91 | 59.8% | 114 | 3.1% | 6 | 0.0% | 0 | 31.1% | 34 | 1.7% | 3 |
| 24 | 41% | 2289 | 75% | 1754 | 35% | 812 | 35.4% | 287 | 52.5% | 426 | 30.1% | 247 | 3.2% | 31 | 1.1% | 2 | 22.7% | 84 | 5.6% | 36 |
| 25 | 35% | 1949 | 69% | 1406 | 37% | 219 | 36.5% | 270 | 53.6% | 397 | 36.8% | 252 | 7.8% | 44 | 2.7% | 17 | 24.9% | 86 | 2.0% | 15 |
| 26 | 19% | 1710 | 67% | 1250 | 35% | 642 | 59.8% | 384 | 71.4% | 459 | 25.0% | 160 | 2.6% | 17 | 0.0% | 0 | 31.2% | 50 | 7.3% | 47 |
| 27 | 27% | 1111 | 73% | 836 | 45% | 519 | 48.1% | 250 | 52.4% | 272 | 46.0% | 239 | 6.4% | 33 | 0.4% | 2 | 13.7% | 31 | 3.1% | 16 |
| 28 | 32% | 1231 | 80% | 1108 | 41% | 572 | 50.1% | 286 | 56.4% | 323 | 45.2% | 258 | 6.2% | 35 | 1.6% | 9 | 26.9% | 66 | 3.0% | 17 |
| 29 | 39% | 2835 | 72% | 2120 | 41% | 1219 | 66.7% | 813 | 76.7% | 935 | 21.5% | 262 | 3.8% | 46 | 0.8% | 10 | 8.0% | 20 | 2.3% | 28 |
| 30 | 44% | 2156 | 66% | 1452 | 27% | 594 | 33.9% | 201 | 34.0% | 202 | 45.5% | 270 | 13.2% | 78 | 2.9% | 17 | 27.8% | 107 | 3.8% | 23 |
| 31 | 26% | 1239 | 69% | 908 | 31% | 186 | 51.4% | 211 | 62.1% | 255 | 38.4% | 158 | 5.9% | 24 | 0.1% | 0 | 24.3% | 37 | 2.6% | 11 |
| 32 | 52% | 2807 | 66% | 1878 | 28% | 789 | 40.4% | 319 | 59.3% | 468 | 33.1% | 262 | 4.3% | 34 | 0.3% | 3 | 18.7% | 59 | 2.9% | 23 |
| 33 | 31% | 971 | 83% | 839 | 55% | 556 | 6.6% | 37 | 12.0% | 67 | 78.0% | 434 | 11.2% | 62 | 1.2% | 7 | 23.5% | 122 | 1.4% | 8 |
| 34 | 18% | 864 | 54% | 486 | 22% | 198 | 16.0% | 32 | 14.0% | 28 | 36.0% | 71 | 49.9% | 99 | 0.0% | 0 | 15.8% | 26 | 6.6% | 13 |
| 35 | 22% | 2339 | 63% | 1535 | 33% | 804 | 46% | 369 | 51% | 410 | 23.0% | 464 | 10.8 | 75 | 0.9% | 8 | 30% | 124 | 9% | 71 |
| 36 | 22% | 546 | 71% | 426 | 49% | 295 | 26.6% | 78 | 27.9% | 82 | 52.8% | 156 | 18.1% | 53 | 0.0% | 0 | 39.1% | 79 | 2.1% | 6 |
| 37 | 34% | 2074 | 72% | 1556 | 46% | 994 | 55.8% | 555 | 65.0% | 646 | 30.9% | 307 | 2.3% | 23 | 0.1% | 1 | 24.6% | 93 | 12.4% | 123 |
| 38 | 31% | 1494 | 74% | 1152 | 34% | 529 | 35.9% | 190 | 37.0% | 196 | 58.0% | 307 | 4.4% | 23 | 0.2% | 1 | 35.1% | 120 | 6.9% | 37 |
| 39 | 28% | 1551 | 71% | 1135 | 40% | 640 | 53.1% | 340 | 70.0% | 448 | 31.3% | 200 | 2.8% | 18 | 0.3% | 2 | 17.9% | 39 | 9.1% | 58 |
