# Supplementary material for: Immune Dysregulation in Pediatric Common Variable Immunodeficiency: Implications for the Diagnostic Approach
Source: Front Pediatr. 2022 Mar 23;10:855200. doi: 10.3389/fped.2022.855200 (PMC8983883; doi:10.3389/fped.2022.855200)
Supplement: Supplementary file 3 [file Table_3.DOCX]

Supplemental Table 2b. Flow cytometric analysis of Tc cell subpopulations in CVID children

| **Patient No** | **Tc cell subpopulations** | | | | | | | | | | | | | |
| --- | --- | --- | --- | --- | --- | --- | --- | --- | --- | --- | --- | --- | --- | --- |
|  | **CD45** | | **CD3+** | | **CD3+CD8+** | | **CD3+CD8+**  **CD27+CD197+**  **CD45RA+** | | **CD3+CD8+**  **CD27+CD197+**  **CD45RA-** | | **CD3+CD8+**  **CD27-CD197-**  **CD45RA-** | | **CD3+CD8+**  **CD27-CD197-**  **CD45RA+** | |
| 1 | 39% | 3861 | 67% | 2618 | 22% | 861 | 59.3% | 511 | 1.0% | 9 | 3.3% | 29 | 23.9% | 206 |
| 2 | 52% | 5862 | 75% | 4475 | 26% | 1565 | 71.6% | 1120 | 4.7% | 73 | 0.6% | 9 | 1.8% | 29 |
| 3 | 27% | 1340 | 65% | 892 | 17% | 237 | 79.3% | 188 | 2.5% | 6 | 0.5% | 1 | 1.9% | 5 |
| 4 | 28% | 3865 | 47% | 1905 | 23% | 916 | 26.1% | 239 | 2.2% | 20 | 19.0% | 174 | 12.8% | 117 |
| 5 | 39% | 2317 | 65% | 1583 | 23% | 551 | 59.2% | 326 | 3.6% | 20 | 3.7% | 21 | 4.9% | 27 |
| 6 | 33% | 2714 | 68% | 1852 | 17% | 464 | 73.3% | 340 | 0.0% | 0 | 0.0% | 0 | 0.4% | 2 |
| 7 | 43% | 2338 | 69% | 1636 | 24% | 555 | 69.9% | 388 | 3.6% | 20 | 0.5% | 3 | 0.9% | 5 |
| 8 | 30% | 1769 | 68% | 1324 | 20% | 387 | 47.2% | 183 | 7.4% | 29 | 12.3% | 48 | 6.1% | 24 |
| 9 | 44% | 2210 | 75% | 1668 | 31% | 699 | 61.7% | 432 | 6.5% | 46 | 12.5% | 87 | 3.3% | 23 |
| 10 | 31% | 1835 | 64% | 1186 | 29% | 538 | 21.6% | 116 | 3.3% | 18 | 11.0% | 59 | 24.9% | 134 |
| 11 | 37% | 2128 | 68% | 1476 | 22% | 478 | 61.5% | 325 | 4.5% | 25 | 0.6% | 3 | 3.4% | 18 |
| 12 | 33% | 1248 | 65% | 869 | 22% | 295 | 61.6% | 182 | 2.4% | 7 | 3.1% | 9 | 8.6% | 25 |
| 13 | 55% | 1745 | 68% | 1243 | 24% | 444 | 24.0% | 106 | 1.6% | 7 | 5.0% | 22 | 11.5% | 51 |
| 14 | 45% | 2786 | 64% | 1871 | 18% | 536 | 62.5% | 335 | 4.2% | 22 | 0.8% | 4 | 4.4% | 24 |
| 15 | 36% | 1626 | 67% | 1122 | 25% | 413 | 61.6% | 255 | 3.4% | 14 | 1.7% | 7 | 8.4% | 35 |
| 16 | 29% | 2873 | 72% | 2096 | 35% | 1012 | 9.8% | 99 | 15.5% | 156 | 3.5% | 35 | 4.5% | 45 |
| 17 | 34% | 1412 | 58% | 907 | 24% | 378 | 49.8% | 188 | 1.1% | 4 | 1.0% | 4 | 9.0% | 34 |
| 18 | 23% | 1559 | 78% | 1284 | 29% | 482 | 11.2% | 54 | 3.5% | 17 | 40.0% | 193 | 10.7% | 52 |
| 19 | 37% | 2070 | 66% | 1473 | 29% | 653 | 46.7% | 305 | 1.6% | 11 | 8.9% | 58 | 13.4% | 88 |
| 20 | 32% | 1626 | 62% | 1058 | 27% | 454 | 27.7% | 126 | 2.5% | 12 | 36.9% | 167 | 6.8% | 31 |
| 21 | 32% | 1236 | 57% | 711 | 18% | 225 | 46.2% | 104 | 6.1% | 14 | 7.7% | 17 | 1.4% | 3 |
| 22 | 26% | 1451 | 72% | 1117 | 23% | 348 | 35.6% | 124 | 6.4% | 22 | 6.1% | 21 | 5.4% | 19 |
| 23 | 11% | 410 | 75% | 344 | 31% | 143 | 80.7% | 115 | 2.4% | 3 | 1.5% | 2 | 0.0% | 0 |
| 24 | 41% | 2289 | 75% | 1754 | 35% | 809 | 61.2% | 331 | 5.6% | 28 | 4.8% | 23 | 4.0% | 24 |
| 25 | 35% | 1949 | 69% | 1406 | 32% | 652 | 16.5% | 62 | 2.4% | 12 | 22.0% | 48 | 1.8% | 12 |
| 26 | 19% | 1710 | 67% | 1250 | 29% | 535 | 66.9% | 358 | 4.5% | 24 | 3.1% | 17 | 2.9% | 16 |
| 27 | 27% | 1111 | 73% | 836 | 21% | 243 | 44.2% | 107 | 5.9% | 14 | 8.2% | 20 | 9.0% | 22 |
| 28 | 32% | 1231 | 80% | 1108 | 33% | 361 | 21.4% | 99 | 1.2% | 5 | 9.2% | 42 | 52.3% | 241 |
| 29 | 39% | 2835 | 72% | 2120 | 27% | 812 | 43.9% | 357 | 1.9% | 15 | 4.9% | 40 | 17.4% | 142 |
| 30 | 44% | 2156 | 66% | 1452 | 32% | 704 | 24.3% | 171 | 13.8% | 97 | 22.5% | 158 | 10.7% | 75 |
| 31 | 26% | 1239 | 69% | 908 | 23% | 299 | 53.1% | 159 | 2.2% | 7 | 9.6% | 29 | 6.3% | 19 |
| 32 | 52% | 2807 | 66% | 1878 | 31% | 886 | 25.5% | 226 | 2.2% | 20 | 38.2% | 338 | 13.1% | 116 |
| 33 | 31% | 971 | 83% | 839 | 23% | 232 | 26.0% | 60 | 8.8% | 20 | 5.1% | 12 | 32.2% | 75 |
| 34 | 18% | 864 | 54% | 486 | 21% | 189 | 22.3% | 42 | 1.0% | 2 | 29.0% | 55 | 24.8% | 47 |
| 35 | 22% | 2339 | 63% | 1535 | 36% | 877 | 42.6% | 270 | 8.2% | 52 | 27.4% | 174 | 7.4% | 47 |
| 36 | 22% | 546 | 71% | 426 | 17% | 102 | 36.0% | 37 | 6.4% | 7 | 13.5% | 14 | 5.8% | 6 |
| 37 | 34% | 2074 | 72% | 1556 | 18% | 389 | 61.5% | 239 | 7.0% | 27 | 7.7% | 30 | 4.0% | 16 |
| 38 | 31% | 1494 | 74% | 1152 | 35% | 545 | 30.6% | 167 | 7.2% | 39 | 22.3% | 121 | 17.9% | 98 |
| 39 | 28% | 1551 | 71% | 1135 | 23% | 368 | 57.2% | 210 | 12.8% | 47 | 9.5% | 35 | 4.3% | 16 |
