# Supplementary material for: Immune Dysregulation in Pediatric Common Variable Immunodeficiency: Implications for the Diagnostic Approach
Source: Front Pediatr. 2022 Mar 23;10:855200. doi: 10.3389/fped.2022.855200 (PMC8983883; doi:10.3389/fped.2022.855200)
Supplement: Supplementary file 4 [file Table_4.DOCX]

Supplemental Table 3. Flow cytometric analysis of NK cells in CVID children

| **Patient No** | **NK cells** | | | |
| --- | --- | --- | --- | --- |
|  | **CD45** | | **CD3- CD16+CD56+** | |
| 1 | 39% | 3861 | 16% | 654 |
| 2 | 52% | 5862 | 17% | 118 |
| 3 | 27% | 1340 | 8% | 115 |
| 4 | 28% | 3865 | 19% | 754 |
| 5 | 39% | 2317 | 14% | 350 |
| 6 | 33% | 2714 | 9% | 244 |
| 7 | 43% | 2338 | 6% | 144 |
| 8 | 30% | 1769 | 12% | 224 |
| 9 | 44% | 2210 | 7% | 158 |
| 10 | 31% | 1835 | 18% | 334 |
| 11 | 37% | 2128 | 7% | 152 |
| 12 | 33% | 1248 | 15% | 202 |
| 13 | 55% | 1745 | 7% | 124 |
| 14 | 45% | 2786 | 18% | 522 |
| 15 | 36% | 1626 | 8% | 139 |
| 16 | 29% | 2873 | 21% | 597 |
| 17 | 34% | 1412 | 20% | 309 |
| 18 | 23% | 1559 | 1% | 20 |
| 19 | 37% | 2070 | 6% | 128 |
| 20 | 32% | 1626 | 8% | 134 |
| 21 | 32% | 1236 | 20% | 251 |
| 22 | 26% | 1451 | 8% | 119 |
| 23 | 11% | 410 | 6% | 26 |
| 24 | 41% | 2289 | 10% | 239 |
| 25 | 35% | 1949 | 10% | 204 |
| 26 | 19% | 1710 | 5% | 95 |
| 27 | 27% | 1111 | 9% | 107 |
| 28 | 32% | 1231 | 7% | 93 |
| 29 | 39% | 2835 | 6% | 169 |
| 30 | 44% | 2156 | 18% | 396 |
| 31 | 26% | 1239 | 14% | 186 |
| 32 | 52% | 2807 | 22% | 613 |
| 33 | 31% | 971 | 9% | 91 |
| 34 | 18% | 864 | 22% | 198 |
| 35 | 22% | 2339 | 8% | 195 |
| 36 | 22% | 546 | 10% | 58 |
| 37 | 34% | 2074 | 5% | 108 |
| 38 | 31% | 1494 | 9% | 140 |
| 39 | 28% | 1551 | 12% | 192 |
