# Supplementary material for: Immune Dysregulation in Pediatric Common Variable Immunodeficiency: Implications for the Diagnostic Approach
Source: Front Pediatr. 2022 Mar 23;10:855200. doi: 10.3389/fped.2022.855200 (PMC8983883; doi:10.3389/fped.2022.855200)
Supplement: Supplementary file 5 [file Data_Sheet_1.DOCX]

**Cell preparation and peripheral blood flow cytometric immunophenotyping**

Peripheral venous blood samples anticoagulated with ethylenediaminetetracetic acid (EDTA-K_2_) were stored at a temperature of between 4 and 8°C and processed within 24 hours. Cells were labelled with the following murine fluorochrome-stained monoclonal antibodies: anti-CD45 FITC (fluoresceine isothiocyanate), anti-CD14 PE (phycoerithrin), anti-CD19 PE, anti-CD19 PerCP (peridinin chlorophyll protein), anti-IgM FITC, anti-IgD FITC, anti-CD38 APC (allophycocyanin), anti-CD27 PE, anti-CD21 FITC, as well as anti-CD3 FITC, anti-CD4 FITC, CD45RA FITC, CD127 FITC, CD185 FITC, anti-CD8 PE, anti-CD16+CD56 PE, CD25 PE, CD31 PE, CD45RO PE, anti-CD3 PerCP, CD197 PerCP, anti-CD4 APC and anti-CD8 APC (all Beckton-Dickinson Biosciences, USA).

Blood samples were mixed with antibodies, incubated in a lysing solution (FACS Lysing Solution, Beckton-Dickinson, USA), centrifuged twice, and suspended in a phosphate buffered saline (PBS, Roche, Germany). The acquisition of cells and analysis were carried out with the use of the flow cytometer FACSCanto and FACSDiva software (Beckton-Dickinson, USA). With sequential gating on biparametric scattering CD45+CD14- lymphocytes, the following lymphocyte subpopulations were identified:

- CD19+ B cells, immature CD19+CD21lo, immature activated CD19+CD38loCD21lo, transitional CD19+CD38hisIgMhi, non-switched memory CD19+CD27+sIgD+, switched memory CD19+CD27+IgD- B cells, and CD19+CD38hisIgM- plasmablasts
- CD3+ T cells, CD3+CD4+ T helper cells, CD3+CD4+CD31+CD45RA+ recent thymic emigrants, naïve CD3+CD4+CD27+CD45RA+, regulatory CD3+CD4+CD25++CD27-, central memory CD3+CD4+CD27+CD45RO+, effector memory CD3+CD4+CD27-CD45RO+, terminally differentiated CD3+CD4+CD27-CD45RA+, follicular CD3+CD4+CD185+CD45RO+, and regulatory CD3+CD4+CD45RO+CD127-CD25++ T helper cells. Among CD3+CD8+ cytotoxic T cells, the following subsets were distinguished: naïve CD3+CD8+CD197+CD27+CD45RA+, central memory CD3+CD8+CD197+CD27+CD45RO+, effector memory CD3+CD8+CD197-CD27-CD45RO+, and terminally differentiated CD3+CD8+CD197-CD27-CD45RA+ cells.
- CD3-CD16+CD56+ NK cells

The relative values of peripheral blood lymphocytes, the B, T, and NK cells of the total lymphocyte population as well as B and T cell subsets were calculated. The absolute counts of all cell subsets were calculated from the peripheral blood leukocyte counts. A comparative analysis was done with the peripheral blood B and T lymph cells immunophenotyping carried out by Piatosa et al [21] and Schatorje et al [22] and served to elaborate the reference cut-off values for pediatric populations at different age groups.
